# Facility-Based Audit System With Integrated Community Engagement to Improve Maternal and Perinatal Health Outcomes in Rural Pakistan: Protocol for a Mixed Methods Implementation Study

**DOI:** 10.2196/49578

**Published:** 2023-11-30

**Authors:** Zahid Memon, Wardah Ahmed, Shah Muhammad, Sajid Soofi, Shanti Chohan, Arjumand Rizvi, Paul Barach, Zulfiqar A Bhutta

**Affiliations:** 1 The Aga Khan University Karachi Pakistan; 2 Jefferson College of Population Health, Thomas Jefferson School of Medicine, Sigmund Freud University Vienna Austria

**Keywords:** audit system, perinatal outcome, neonatal mortality, stillbirth, maternal, mortality, implementation research, death audit

## Abstract

**Background:**

Maternal and newborn mortality in Pakistan remains as a major public health challenge. Pakistan faces significant infrastructure challenges and inadequate access to quality health care, exacerbated by sociocultural factors. Facility-based audit systems coupled with community engagement are key elements in achieving improved health system performance. We describe an implementation approach adapted from the World Health Organization audit cycle in real-world settings, with a plan to scale-up through mixed methods evaluation plan.

**Objective:**

This study aims to implement a locally acceptable and relevant audit system and evaluate its feasibility within the rural health system of Pakistan for scale-up.

**Methods:**

The implementation of the audit system comprises six phases: (1) identify facility and community leadership through consultative meetings with government district health offices, (2) establish the audit committee under the supervision of district health officer, (3) initiate audit with ongoing community engagement, (4) train the audit committee members, (5) launch the World Health Organization audit cycle (monthly meetings), and (6) quarterly review and refresher training. Data from all deliveries, live births, maternal deaths, maternal near misses, stillbirths, and neonatal deaths will be identified and recorded from four sources: (1) secondary-level care rural health facilities, (2) lady health workers’ registers, (3) community representatives, and (4) project routine survey team. Concurrent quantitative and qualitative data will be drawn from case assessments, process analysis, and recommendations as components of iterative improvement cycles during the project. Outcomes will be the geographic distribution of mortality to measure the reach, proportion of facilities initiated to implement an audit system for measuring the adoption, proportion of audit committees with community representation, and proportion of audit committee members’ sharing feedback regularly to measure acceptability and feasibility. In addition, outcomes of effectiveness will be measured based on data recording and reporting trends, identified modifiable factors for mortality and morbidity as underpinned by the Three Delays framework. Qualitative data will be analyzed based on perceived facilitators, barriers, and lessons learned for policy implications. Results will be summarized in frequencies and percentages and triangulated by the project team. Data will be analyzed using Stata (version 16; StataCorp) and NVivo (Lumivero) software.

**Results:**

The study will be implemented for 20 months, followed by an additional 4-month period for follow-up. Initial results will be presented to the district health office and the District Health Program Management Team Meeting in the districts.

**Conclusions:**

This study will generate evidence about the feasibility and potential scale-up of a facility-based mortality audit system with integrated community engagement in rural Pakistan. Audit committees will complete the feedback loop linking health care providers, community representatives, and district health officials (policy makers). This implementation approach will serve decision makers in improving maternal and perinatal health outcomes.

**International Registered Report Identifier (IRRID):**

DERR1-10.2196/49578

## Introduction

### Background

Pakistan is a low-income country with significant infrastructure challenges, poor allocation of government funding for health, poor access to essential health care services, low-quality health care services [1], sociocultural constraints, and a feudal system that affects all aspects of province life [2]. One of the main impacts of these governance and funding challenges relates to the health of mothers and their newborn children—2 cohorts that are already categorized as populations at high risk. The manifestations of this burden include a persistently high neonatal mortality rate, accounting for 57% of child deaths across the nation [3], with 41 out of every 1000 born babies dying before the end of their first month [4] and a maternal mortality ratio of 186 per 100,000 live births [5]. Pakistan’s perinatal, neonatal, infant, under 5 years, and maternal mortality rates remain unacceptably high, with estimates of >399,429 children dying per year [6]. Although efforts to improve the quality and timeliness of care in district and regional facilities are being made, progress has been painfully slow [7].

Audits and review systems, especially those with community engagement, are 2 key elements that have been shown to improve the health system’s performance and maternal and perinatal health outcomes for at-risk populations [8]. Audits provide a documented history of the events leading up to the death and highlight process failures amenable to process redesign and teachable moments to prevent similar untoward events. Introducing audits and reviews can reduce inpatient maternal mortality (adjusted odds ratio 0.85, 95% CI 0.73-0.98; 191,167 deliveries; moderate certainty evidence) and likely inpatient neonatal mortality (adjusted odds ratio 0.74, 95% CI 0.61-0.90; moderate certainty evidence) [9]. Findings from a meta-analysis of 7 pre-post studies of facility-based perinatal mortality audits in low- and middle-income countries indicated a reduction in perinatal mortality of 30% (95% CI 21%-38%) after introduction of perinatal audits [10].

The District Health Information System (DHIS) seldom records and reports mortality (eg, maternal mortality and neonatal mortality) and severe morbidity indicators consistently and reliably (eg, birth asphyxia and preeclampsia), leading to challenges in data completeness in capturing vital statistics through routine health and administrative data. Audit systems at the facility levels can provide for sustained improvements in the quality of provision and delivery of services. Community linkages and engagement ensure proactive and detailed discussion of the causes and modifiable factors leading to the deaths, essential for improved working relationships between community members, health providers, and policy makers [11]. Audits of stillbirths and neonates also contribute to achieving Every Newborn Action Plan goal for reducing stillbirths and neonatal deaths to <12 newborn deaths per 1000 live births [12].

### The Three Delays Framework for Maternal and Perinatal Mortality

Pregnancy-related maternal, neonatal, and infant mortality is attributed to delays in using the Three Delays framework [13] with time-related consequences—the “first delay” around the decision to seek medical care, “second delay” attributed to the time to reach the health facility, and “third delay” caused by receiving inappropriate care after reaching the medical facility [14-16]. In Pakistan, the “first delay” has been further divided into two subgroups: (1) delay in recognition of the severity of illness and danger signs contributed to 18% of all delays and (2) delays in decision-making by virtue of limited action of women to make decisions regarding their transfer to facilities contributed 34% [7].

The impacts of delays in care can be seen in the recent Verbal Autopsy and Social Autopsy mortality survey in Table 1. More than half of the deaths (26%-55%) occurred at the community level (the first delay), 4% to 13% of deaths occurred in route to the facility (the second delay), and the remaining occurred within health care systems (the third delay).

**Table 1:** Locations of neonatal, child, and maternal deaths according to the unpublished Verbal Autopsy and Social Autopsy results (2019).

Indicators	Hospital, %	Other health provider or facility, %	In route to health provider or facility, %	Home, %	Others, %
Location of neonatal deaths	55.3	2.3	4.1	38.2	0
Location of child deaths	35	0.9	7.1	54.7	2.2
Location of maternal deaths	56.8	11.4	12.5	26.4	3

### History of Perinatal Audit Systems in Pakistan

Previous efforts to introduce facility audit systems by the Pakistan Ministry of National Health Services, Regulations, and Coordination have used the phased implementation of the Maternal and Perinatal Death Surveillance and Response to enhance reporting, recording, tracking, and auditing of deaths [17]. The World Health Organization (WHO) is currently assisting Khyber Pakhtunkhwa and Baluchistan provinces, and United Nations International Children’s Emergency Fund (UNICEF) is helping the Sindh province in implementing audit mechanisms, but results have not been made public. The United Nations brief on COVID-19 [18] and the Pakistan Health Report 2020 [19] have stated that the Maternal and Perinatal Death Surveillance and Response has great potential for success.

We hypothesize that a WHO audit cycle that is locally acceptable in close consultation with local community representatives and health care providers is feasible for implementing a robust audit system in rural settings. Specifically, we asked the following questions:

1. Is the implementation of facility-based maternal and perinatal mortality audits in combination with targeted community engagement feasible in rural Pakistan?
2. Will the maternal and perinatal audits improve the reporting of deaths and maternal near misses at the DHIS health facilities?
3. Will the audit systems help better identify medical and nonmedical factors of deaths and near misses, and how doable recommendations are implemented through integration of feedback loops within the health facilities and communities?
4. Will the community-facility audit interaction improve the early referral proportions?

### Primary Objectives

The primary objectives include the following:

1. Identify and establish audit committees at health facility levels with integrated community engagement, and
2. Determine the feasibility of implementing an acceptable audit system with community representation in existing secondary health facilities and serving catchment populations.

### Secondary Objectives

The secondary objectives include the following:

1. Identification of “first delay” (care-seeking decision), “second delay” (identification and reaching the health facility), and “third delay” (receiving adequate care and treatment at facilities) among mortality and morbidity cases, and
2. Propose solutions and record actions taken to improve the access to quality of care through integrated feedback loops within health facilities and communities.

### Primary Outcomes

The primary outcomes include the following:

1. Proportion of facilities initiated to implement an audit system for measuring the adoption of audit system,
2. Proportion of audit committees with community representation and proportion of committee audit members sharing feedback regularly to measure the feasibility of the audit system, and
3. Qualitative narrative approach for acceptability and exploring the facilitators of and challenges in implementing audit systems.

### Secondary Outcomes

The secondary outcomes include the following:

1. Geographic distribution of mortality to measure the percentage coverage or reach in the implementation district,
2. Change in trends in data recording and reporting to measure effectiveness,
3. Categorization of mortality and maternal near-miss cases among Three Delays framework,
4. Number of awareness sessions covering the proportion of the catchment population,
5. Number of referrals in and out of the health facility,
6. Number of transport mechanisms linked to health facilities by audit committee members, and
7. Proportion of health facilities implementing actionable recommended actions at the facility level.

## Methods

### Overview

We will conduct a mixed methods study that integrates formative research and evaluative components into iterative improvement cycles. The planned interventions will examine the feasibility and document the process and challenges of implementing an audit system at scale. The study was also registered on ClinicalTrials.gov (NCT05640050).

### Study Setting

The study will be conducted in 1 district—Matiari (Sindh) with a population of 770,040 (Pakistan Bureau of Statistics–2017). The district selection was based on the operational ease with past positive experiences in efficient rollout of health services. Moreover, the district health office (DHO) of Matiari is actively involved in DHIS feedback meetings to improve the data quality.

### Study Population

The eligible population includes all women who had deliveries at community and health facilities that result in a maternal death or maternal “near miss,” stillbirths, or neonatal death (perinatal death).

### Inclusion Criteria

Women aged between 15 and 49 years who reside in the selected district are eligible to participate. Secondary-level public health facilities that offer obstetric and postnatal care and respective catchment areas supported by lady health workers (LHWs) are included in the study. Data for all maternal mortality, maternal “near misses,” and perinatal and neonatal mortality recorded for all women and newborns delivered at home (through LHW monthly reports) and who contact the health facility within 42 days after delivery, regardless of whether they delivered at the health facility, will be collected.

### Exclusion Criteria

Women who are nonresidents of the study district and who do not provide consent are not eligible to participate.

### Operational Definitions

The outcomes are defined in Textbox 1, using the International Classification of Disease–10 classification system by WHO [20].

**Operational definitions—International Classification of Diseases–10.**

**Stillbirth**

- Stillbirth is the delivery of baby with no signs of life after ≥22 weeks of gestation.

**Neonatal mortality**

- Neonatal mortality is the death of a newborn within the first 28 days of life.

**Perinatal mortality or death**

- Perinatal mortality or death is a death that occurs around the time of birth. It can involve a still birth or be an early neonatal death that occurs up to 7 days after birth.

**Maternal *near miss***

- Maternal *near miss* is a situation in which a pregnant woman nearly dies but survives a complication that occurred during pregnancy, during childbirth, or within 42 days of the termination of pregnancy. It can involve severe pregnancy-related complications (severe postpartum hemorrhage, severe pre-eclampsia, eclampsia, sepsis, and ruptured uterus) or critical interventions (blood transfusion, laparotomy, admission to an intensive care unit, hysterectomy, and other emergency surgical interventions in the abdominal cavity).

**Maternal mortality or death**

- Maternal mortality or death is the death of a woman during pregnancy or within 42 days of delivery or the termination of pregnancy, irrespective of the duration or site of the pregnancy, from any cause related to or aggravated by the pregnancy or its management, but not from accidental or incidental causes.

### Sample Size

Population size is a challenge in the rural setting, however this study intend to cover all records of stillbirths, neonatal deaths, maternal deaths, and maternal “near misses” from four sources: (1) secondary health facilities, (2) LHW registers, (3) audit committee’s community representatives, and (4) project routine survey team.

### Study Timeline

**Figure 1:**
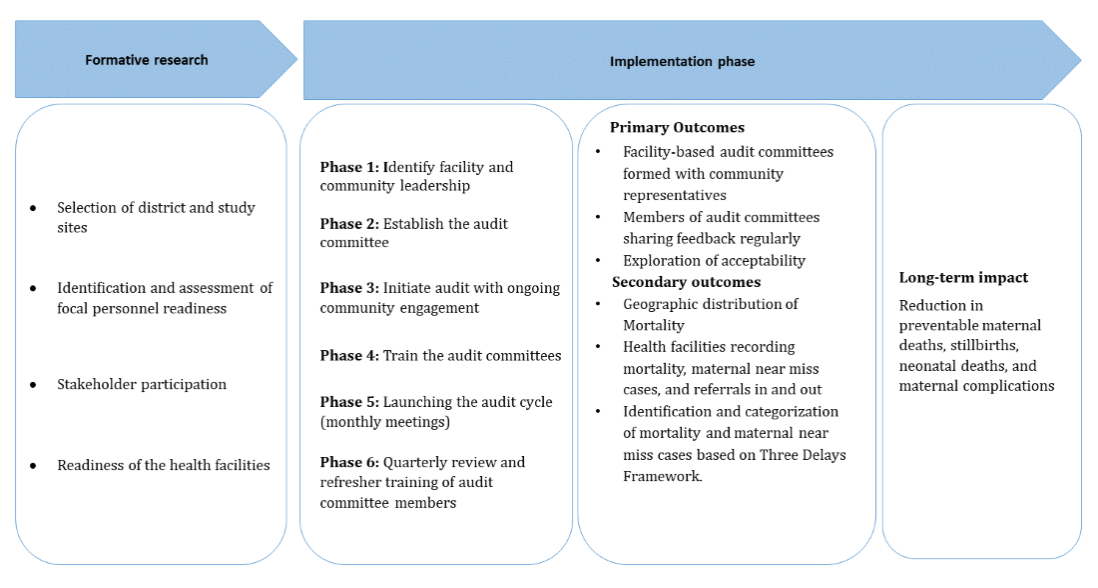
Implementation framework.

The implementation of the audit system intervention is planned for 20-month period. To account for the effects of maturation (ie, 28 days after delivery for neonatal outcomes and 42 days after pregnancy for maternal near-miss outcomes), an additional 4 months will be added to the end of the intervention. Figure 1 Illustrates the implementation framework.

### Audit System Implementation Phases (6 Phases)

The project development is based on formative studies that identified the importance of health care providers’ readiness, stakeholder and community participation, and health facilities’ readiness. The process will include consultative meetings with project’s core committee members, program advisory committee members, Director General of Health, and DHO staff.

#### Phase 1: Identify Facility and Community Leadership

The project’s core team and district manager identify facilities and community representatives who will coordinate and liaise with other district stakeholders to implement the community audit interventions.

#### Phase 2: Establish the Audit Committee

The person in charge of the health facility will establish an audit committee using existing health committees at the Tehsil Head Quarters, quality control committees, medical inspection committees, health welfare committees, and joint health inspection committees in health facilities at Matiari. Audit committees will be led by the medical superintendent of the hospital, with a member from administration, 3 to 4 consultants or medical officers from various departments (gynecologist; pediatrician; ear, nose, and throat [ENT] specialist; pathologist; head of the emergency department; and head nurse), district magistrate, or focal person from DHOs. The community engagement and audit system for project audit committee will be established at each facility; preferably, members will be at least 2 obstetricians, 2 pediatricians, 1 administrator, project focal person, and community representatives as community audit representative. Responsibilities and audit committee structure will be described. Members of the audit committee will update their credentials every month, and trickle-down training will be conducted by audit committee members.

#### Phase 3: Initiate Audit With Ongoing Community Engagement

Community representatives (1-3 individuals) will be identified as community audit representatives. They will be invited to attend the monthly audit meetings with facility-based audit committee members to discuss the community perspectives (delays 1 and 2) and other issues pertaining to the negative maternal and perinatal outcomes. LHWs, lady health visitors, community midwives (CMWs), male community mobilizer (village or otaq leader or any other active member of village), or any other influential women in the village will be engaged for audit committee decisions. They will provide timely oversight, monitor, and respond to adverse events and later confer in the audit meetings.

#### Phase 4: Train the Audit Committees

Training for audit representatives and members of the audit committee, will be conducted using 3-day training workshop, which includes the following:

- Day 1: Maternal death, near misses, and perinatal death (stillbirth and neonatal deaths),
- Day 2: The Three Delays framework and identifying modifiable factors, and
- Day 3: Mentoring regarding the identification of the audit committees and initiating the audit implementation committee.

The audit committee will be trained to follow ethical guidelines in maintaining an empathetic gesture while recalling the adverse patient events by the respondents. Training will also include standardized case studies and completion of audit meeting minutes and action item tools.

#### Phase 5: Launch the Audit Cycle (Monthly Meetings)

##### Overview

**Figure 2:**
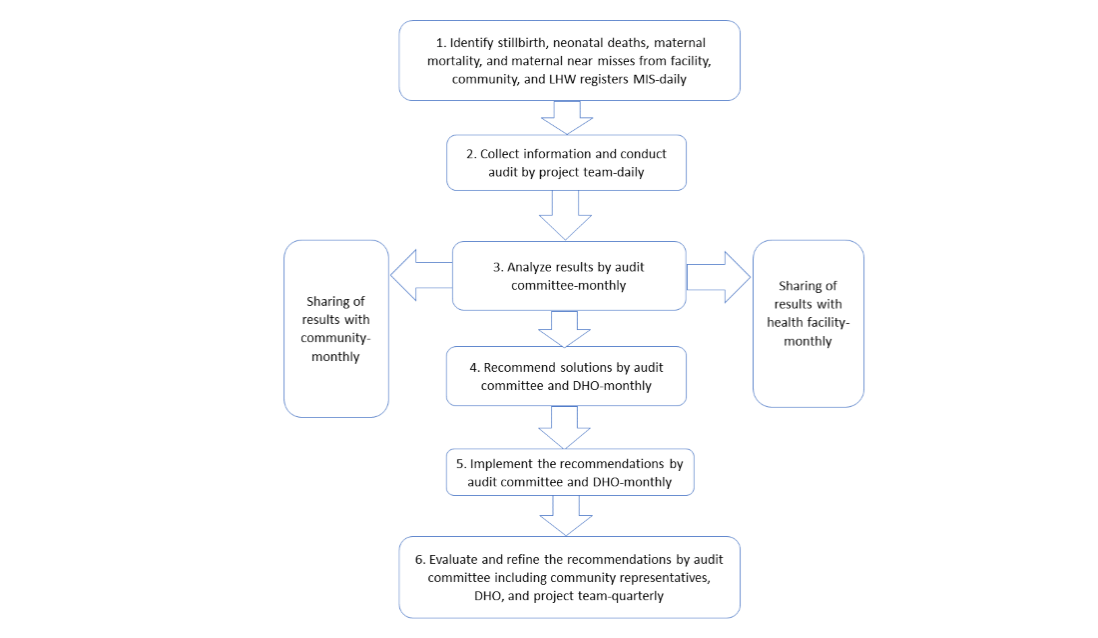
World Health Organization audit cycle. DHO: district health officer; LHW: lady health worker; MIS: management information system.

The WHO audit system [20] will be adapted to assess maternal, perinatal, and neonatal deaths with 6 steps. Audit process in health facilities and communities is illustrated in Figure 2. Following are the steps and their processes involved in audit cycle.

##### Step 1: Identifying Cases for Review

The district supervisors will record all maternal deaths, maternal near misses, deliveries, births, stillbirths, and neonatal deaths that occur in the delivery ward, neonatal unit, and postnatal ward, ensuring the capture of the minimum set of perinatal indicators in a source register. Efforts to collect and record data on maternal deaths, near misses, and perinatal and neonatal deaths that occur in the community will be made through monthly reporting from LHWs or CMWs to their associated health facility or by women who contact the health facility after delivery. Specific strategies will be put in place to ensure that all deaths are captured and filtered to the mortality audit committee, so that cases can be selected for review and discussion at the mortality audit meetings.

##### Step 2: Collecting Information

The aim is to empower designated staff to collect a standardized set of information from the patient file or register as soon as possible after the death. For every death, decisions will be taken regarding what information is recorded, where the information is recorded, who records it, and who collates it on a periodic basis both for the death review process and for reporting to other levels within the health system. It should be noted that if the use for the data cannot be identified, the data will not be collected. Before collecting the information, the audit committee will have a clear understanding of the data analysis plan to help identify the data to collect. Data abstraction or collection forms will be either electronic or paper-based, depending on the infrastructure and capacity of the health facility. Clear directions about the exact data and qualitative information that should be collected will be included.

All data collected will be stored on password-protected laptops or tablets or locked cabinets in locked offices within the health facility. Before implementation, data collection forms will be piloted and revised as needed. The minimum data collected will include the following: information about the mother’s condition, baby’s condition on admission and at the onset of labor (for antepartum and intrapartum deaths), baby’s condition at birth (stillbirth or live birth), baby’s condition on discharge from the health facility (alive, transferred to another facility, or dead], and date and time of birth and death (so that the age at death can be calculated in hours rather than days).

As part of the intervention, each facility audit system is planned to be comprehensive to ensure that it captures data on each death and includes the relevant cause of death; link to maternal condition; demographic data; and a list of contributing, modifiable factors corresponding to codes for analysis and linked to recommendations. To ensure that phase-1 and phase-2 delays are captured, all efforts to link deaths that occur in the community to the facility audit system will be conducted by the trained LHWs, CMWs, and project team. The audit committee will hold follow-up monthly meetings with designated community engagement members to initiate the chain of notifications within the audit cycle as it relates to the implementation of recommendations and solutions.

##### Step 3: Analyzing the Information

The aim is to identify problems in the system that may have contributed to maternal death, stillbirths, and neonatal deaths, especially those that could have been averted. Data analysis will include quantitative components, such as identification of trends in rates and causes of death and geographic location. Qualitative analysis will provide additional insight into the problems that caused the deaths of individual cases and more general information about groups of deaths caused by similar contributing factors. The use of both types of data together will provide a more robust analysis of the problems and aid the audit committee to identify priorities for action.

##### Step 4: Recommending Solutions

The aim of this critical step is to find ways to move from problems to action by identifying specific, measurable, attainable, relevant, and time-bound solutions. A formal platform will be created to present the findings of the audit process. The moderator will guide the discussion and present aggregated data and trends in addition to selected individual cases presented anonymously without bias. It is recommended that no more than 2 cases per 2-hour session shall be reviewed per month. After the presentation of the case summary, the main facts are analyzed by the audit members. Discussion will follow the presentation, reflecting on the modifiable factors of specific cases and any changes in trends from meeting to meeting.

A systematic and exhaustive analysis of the case should be conducted to effectively understand the chain of events related to the case. After identifying the main problems at the various steps described previously, the audit committee is invited to discuss and make the case for why a procedure or an act should be considered adequate or inadequate, by referring to the established standards of practice. Dysfunctions and their causes should be prioritized, and energy should be devoted to solving the most significant problems and those for which the implementation of a solution is feasible. Then, the committee will investigate what its causes and related factors may be, and these can be related to (1) personnel: qualification, skills, availability, attitude, and so on; (2) drugs, supplies, and equipment: availability, accessibility, and so on; (3) protocols: availability, knowledge, understanding, use, and so on; (4) management or care organization: coordination, communication, and so on; and (5) patient and his family: financial accessibility, misunderstanding, willingness, and so on. Final step will be the identification of cause of morbidity or death by (1) reviewing the medical cause of severe morbidity or death and identifying the various factors or events that may have contributed to the outcome, (2) comparing the cause of the morbidity or death documented in the patient’s record with the audit committee findings, and (3) determining whether the death was preventable.

Following the abovementioned steps in analyzing the case, the moderator leads the discussion to select the problems to be solved in order of priority. Problems must be prioritized based on the significance of their effect on prognosis and the feasibility of the actions necessary to solve them. Through the moderator, the audit committee will then reach a consensus on appropriate, evidence-based strategies to address the main gaps at the health facility level (phase-3 delay). The solutions will balance the priorities based on burden and feasibility and may be related to ongoing or one-off activities.

In addition to possible solutions at the health care facility level, the audit committee in the community will identify cases that are closely linked to the community and agree on select cases to present during the postaudit community feedback meeting. Community-level solutions will be directly related to phase-1 and phase-2 delays. The audit committee will ensure that findings are shared in the appropriate forum and work with the community to develop solutions that are clear, attainable, and straightforward about the potential for improving care, without placing blame.

Recommending realistic solutions to reduce the factors associated with maternal, perinatal, and neonatal morbidity and mortality in the community can be challenging. Careful considerations regarding the capacity of the community to implement the recommendation and the method of communication will be prioritized. The 2016 WHO guidelines for audit and review of stillbirths and neonatal deaths advises formulating a recommendation in collaboration with community leaders empowered to make the recommended change to ensure it is effective. To alleviate the risk of developing distrust between the community and the health system, recommendations will not be “handed down” from the health facility audit committee but rather discussed as a partnership with the community. In addition, effective communication, relayed consistently and sensitively, will be developed through the early selection of appropriate community representatives to participate in the audit meetings. Through the deliberate selection of key community representatives, efforts will be made to encourage an open, synergetic, and nonjudgmental forum between the community and health facility to ensure that the chosen recommendation is attainable and will ultimately strengthen the community voice.

Our data suggest that the process of fostering active partnerships between health care providers and community members through dialogue, planning, and collective action by learning from what went wrong and emerging lessons can improve community trust and quality of care [21].

##### Step 5: Implementing Changes

The aim is to take immediate, medium-term, or long-term actions to prevent maternal deaths, stillbirths, and neonatal deaths, using successes to advocate for and spur further action. Implementing changes is the primary objective of the audit cycle. To be more effective, the audit committees will focus on the modifiable factors that are within the control of health workers and the community.

The findings of the audits will be shared with the concerned authorities in health facilities, program managers, and community representatives to spur policy and practice related change. DHO will be onboard to ensure that the decisions agreed upon are implemented at the facility and community levels. The decisions proposed by the audit committee and endorsed by DHO will be implemented to address the bottlenecks at the demand side (delay in care and financial and mobility challenges) and the supply side (lack of hospital supplies, staff, and resources).

##### Step 6: Monitoring and Evaluation

In addition to the monthly audits, the project committee will also collect administrative and survey data to capture the process and outcome indicators. From independent, third-party surveys and data sources such as DHIS, facility registers, and LHW management information system, the impact of the intervention will be evaluated based on the maternal perinatal and neonatal mortality and morbidity outcomes and rated for all pregnant women, mothers, and newborns.

The model will contain audit guidelines, protocols, and training materials and tools for effective monitoring at the facility level in the targeted districts with their respective catchment populations. A monthly monitoring cycle will be set up within the implementing facilities to ensure effective implementation of the audit systems.

#### Phase 6: Quarterly Review and Refresher Training of Audit Committee Members

For a sustainable audit system, recognition and reinforcement is inevitable. Refresher training and recertification will be conducted quarterly.

### Data Management and Analysis

The data collection tool will be developed in English and then translated into the local language to be administered to the local community visiting the health facilities. Data will be collected manually and entered through a specifically designed application. The application will be installed on laptop or desktop at each health facility under the supervision of project leadership. The Aga Khan University Data Management Unit will develop an electronic database that includes the filters and data quality check indicators. Data will be entered at the end of every week and uploaded onto the Aga Khan University Data Management Unit server. All data files will be stored for 5 to 7 years and then deleted according to organizational procedures for the permanent destruction of electronic and paper data.

Descriptive statistics will be used to estimate maternal, neonatal, and other key quantitative variables. The Three Delays framework will be used to categorize the identified modifiable factors. Action reports will be submitted based on the recommendations proposed by the audit committee. The data will be analyzed using Stata (version 16; StataCorp) and NVivo (Lumivero) software and password-protected Excel (Microsoft Corp) files. A unique code identifier will be assigned for the cases to be selected for discussion in audit meetings. The village name and health facility name will be kept confidential in audit meetings and, instead, codes will be used. In addition, regular meetings, planned dissemination of study findings including manuscript publication, stakeholder knowledge sharing workshop including community participation, and conference presentations will be conducted.

### Ethical Considerations

The study was approved by the ethical review committee of Aga Khan University (ethical review committee number 2021-6426-19638). The project committee will be trained in conducting human subject research keeping in mind the ethics guidelines and respecting the autonomy of the participants. Using a noncoercive attitude, informed consent and maintenance of privacy will be assured while collecting data. Respondents will be asked to participate voluntarily after giving written informed consent. Respondents will be free to ask any questions or may ask to skip any question or may withdraw from interview at any moment without incurring any penalties.

## Results

The study implementation will be for 20 months, followed by an additional 4-month period for follow-up. Initial results will be presented to DHO and the District Health Program Management Team Meeting in the districts.

## Discussion

### Expected Outcomes

The study introduced a research protocol that outlines an intent to establish a systematic audit system for recording, assessing, and evaluating maternal deaths, near misses, stillbirths, and neonatal deaths in rural Pakistan. Previous endeavors of similar nature have been conducted in high-income countries and various low- and middle-income countries [22]. These initiatives underscore the significance of executing a thorough audit cycle, involving effective leadership, engaging relevant health care practitioners, and using participatory methods within communities. Targeting all cases through mortality audits to enhance the standard of care for mothers and newborns. Hence, this subject holds considerable importance for Pakistan’s health care system, particularly for this type of project, which has not been pursued previously to enhance feasibility, continuity, and sustainability.

### Risk Management

There are minimal risks associated with this study. Community members and health care providers may experience some psychological and social risks associated with recalling the consequences surrounding a death. In the event a woman becomes emotionally distressed during the survey, participants will be provided the opportunity to stop and take a break or discontinue the interview completely. Similarly, through the identification and discussion of causes resulting in specific deaths, there is the risk of blaming and shaming of potential health facility staff or community members. All efforts to allay these risks and the repercussions will be made by ensuring that all names of the health care staff who provided care and patients remain anonymous.

The goal of the audit approach is to identify why a death or severe outcome occurred, so that changes can be made to prevent similar events in the future. The purpose is not to cast blame or punish individuals, groups, or institutions. Audit committee members will be encouraged to develop a code of practice to foster an environment of collaboration rather than blame. Accountability for adverse actions will be encouraged through approaches that seek to improve care by educating both the health care provider and community. There is the potential risk that given the size of a catchment area, community representatives may have immediate knowledge about the case under discussion. To create an environment for change in the community, in addition to anonymizing all providers and patients, community representatives will be strategically selected to ensure that they are well respected and have strong, effective communication skills. As the primary factors related to patient-level, family-level, or community-level delays 1 and 2 are associated with knowledge about danger signs and plans for delivery, such as transportation, there is minimal risk associated with individual blaming or shaming. Approaches for implementing the recommendations will be enhanced through the form of media favored by the community, such as radio, television, theater, and murals, in addition to written materials. No unexpected or adverse events are anticipated as a result of the audit reviews.

### Policy and Practice Change Implications of the Audit System

Initially, based on the advice of the district health officer, the meetings will be conducted monthly. Later, dissemination of the implementation study findings will be shared quarterly at the District Health Program Management Team Meeting. This meeting comprises key stakeholders including those in charge of health facilities, DHIS focal person, LHW program focal individuals, and other members involved in and contributing to the design, implementation, and rollout of the audit system delivery model. Consistent liaison with these provincial-level stakeholders will allow for the integration of infrastructural changes and practice changes at the facility level, conducive to the effective implementation of the audit system. Conducting audits focused on maternal and perinatal mortality presents an opportunity to implement effective actionable actions for improving the well-being of mothers and children in limited-resource communities. The implementation approach will be used for integration of decision makers, health care providers, and community members, as they are vital components of the comprehensive audit cycle proposal.

### Limitations

Our project is unique with its focus on community engagement and representation integrated into the facility-based audit system. As such, it has strong implications for scale-up and uptake across Pakistan’s regional districts. Nevertheless, the primary limitation of this study is the recording of neonatal and maternal deaths and their complete medical history from health facilities. To overcome this challenge, there will be planned quarterly refresher training for the facility staff regarding source register apprising and DHIS. Moreover, in monthly audit cycles, the cases will be presented by audit committee members including community representatives. This will help support local ownership for facilitating and using existing resources. DHOs will ensure the presence, retention, and participating attitude of audit committee members. The implementation design includes quantitative and qualitative approaches, which can provide strong evidence for the impact generated by the audit system implementation.

### Patient and Public Involvement

The public was not involved in the development of the study design, but they are an integral part of the implementation of this study, and their perspectives and engagement are key components of the study. The key findings will be regularly shared with the public sector authorities.

### Conclusions

The study will generate evidence about the feasibility and potential scale-up of a facility-based mortality audit system with integrated community engagement in rural Pakistan. Audit committees will complete the feedback loop linking health care providers, community representatives, and district health officials (policy makers). Analyzing the burden of deaths in the area and delving into individual cases aid in identifying the causes and modifiable factors to prevent future adverse events. This implementation approach also serves the purpose of establishing a regular dissemination platform for decision makers to prioritize the actionable recommendations for improving maternal and perinatal health outcomes.

## Abbreviations

CMW: community midwife
DHIS: District Health Information System
DHO: district health office
ENT: ear, nose, and throat
LHW: lady health worker
UNICEF: United Nations International Children’s Emergency Fund
WHO: World Health Organization

## Data Availability

The data set will be available upon request. The data will be restored under Aga Khan University data security and retention policy.
